# Molecular typing of clinical and environmental Aspergillus fumigatus isolates from Iran using microsatellites

**DOI:** 10.18502/cmm.7.1.6180

**Published:** 2021-03

**Authors:** Hamid Badali, Tahereh Shokohi, Sadegh Khodavaisy, Maryam Moazeni, Masoumeh Farhadi, Mojtaba Nabili

**Affiliations:** 1 Invasive Fungi Research Center, Communicable Diseases Institute, Mazandaran University of Medical Sciences, Sari, Iran; 2 Department of Medical Mycology, School of Medicine, Mazandaran University of Medical Sciences, Sari, Iran; 3 Department of Medical Mycology and Parasitology, Tehran University of Medical Science, Tehran, Iran; 4 Department of Medical Laboratory Sciences, Faculty of Medicine, Sari Branch, Islamic Azad University, Sari, Iran; ¥ These authors equally contributed to the work

**Keywords:** *Aspergillus fumigatus*, Iran, Microsatellite, Molecular typing

## Abstract

**Background and Purpose:**

Because of the growing incidence of *Aspergillus* infection, typing methods of *Aspergillus* species are increasingly being used. Accordingly, studying the spread and population dynamics of strains isolating from clinical and environment, from a single host to large-scale ecosystems is definitely needed. In the current study, we carried out a genetic analysis of nine microsatellite loci in isolates from different regions of Iran to compare and explore the genetic diversity between environmental and clinical *A. fumigatus* strains.

**Materials and Methods:**

Sixty-six clinical (n=43) and environmental (n= 23) isolates of *A. fumigatus*, have collected from six cities of Iran. All *A. fumigatus* isolates identified based on macroscopic and microscopic characters, the ability to grow at above 45°C, and confirmed using DNA sequencing of the partial b-tubulin gene. Sixty-six *A. fumigatus* isolates were subjected by microsatellite typing using three separate multiplex PCRs with a panel of nine short tandem repeats (STR) to evaluate the genetic relatedness.

**Results:**

The STR typing of 66 *A. fumigatus* isolates revealed 38 distinct genotypes distributed among environmental and clinical isolates. We identified 12 clones including 40 different isolates representing 60% of all isolates tested, which each clone included 2–7 isolates.

**Conclusion:**

The STR typing is considered as a valuable tool with excellent discriminatory power to study the molecular epidemiology and genotypic diversity of *A. fumigatus* isolates. These findings show that the high genetic diversity observed of Iranian *A. fumigatus* isolates with those outside Iran and formed a separate cluster.

## Introduction

*Aspergillus fumigatus* is an everywhere saprophytic mold with a global distribution frequently occurred in human fungal infections ranging from slight allergic reactions, colonization to serious systemic infections. Invasive aspergillosis (IA) is a main reason of infections among immunocompromised individuals with over 200,000 life-threatening fungal infections yearly [ 1 - 4 ]. Remarkably, *A. fumigatus* is the predominant species to be found from cultures of respiratory samples in cystic fibrosis patients with frequency approximately from 6% to 60% [ 5 , 6 ]. The incidence of IA due to *A. fumigatus* infections in susceptible patients has dramatically increased in recent years due to construction, renovation, demolition and excavation activities in hospitals and clinical centers [ 7 , 8 ]. In addition, failure in treatment of infections caused by acquired triazoles resistant *A. fumigatus* isolates have been increasingly reported, recently.[ 9 - 11 ]. *Aspergillus flavus* is another species of *Aspergillus* that is more common in Iran due to tropical and subtropical climatic conditions. Various studies in Iran from clinical and environmental isolates of *A. flavus* have identified using molecular and genotyping techniques [ 12 - 14 ]. Antifungal resistance is a major threat for treatment and prophylaxis of fungal infections in both immunocompetent and immunocompromised host. Resistance to azole can occur in patients who are used azole for long-term treatment for the management of invasive aspergillosis or may acquire from the environment as a consequence of exposure to azole fungicides applied in agriculture [ 2 , 15 - 17 ]. Therefore, the international surveillance network of *A. fumigatus* azole resistance designed a global project to realize how resistance is created in the environment. This project conducts various studies with the aim of maintaining the use of azoles for the production of food and human medicines [ 18 ]. Regarding the upward trend in incidence of *Aspergillus* infection, typing methods of *Aspergillus* species are extremely being used. Accordingly, studying the spread and population dynamics of strains isolating from clinical and environment, from a single host to large-scale ecosystems is definitely needed [ 19 , 20 ]. Nowadays, molecular typing creates new methods for better infection control. Also, it’s useful for infection or colonization of a single patient and determine the population structure of a species and study of the epidemiological association between environmental and clinical isolates. The disadvantages of pervious molecular typing of *A. fumigatus* methods such as (AFLP), multilocus sequence typing (MLST), pulsed-field gel electrophoresis (PFGE), random amplification of polymorphic DNA (RAPD), restriction fragment length polymorphism (RFLP), RNA-induced silencing complex (RISC), repetitive element sequence-based PCR (rep-PCR), single-stranded DNA binding protein (SSDP), and variable number tandem repeat (VNTR) is lack of discriminatory power and poor interlaboratory reproducibility. However, microsatellites were used to determine and analyzed the genetic distances and have demonstrated to be powerful instruments for molecular typing [ 20 - 22 ]. Microsatellites are repeated motifs of about 1–9 non-coding nucleotides fragments. Due to high rates of mutation and variability, microsatellite sequence modifications is particularly useful for studying differences between closely related species [ 19 , 23 ]. Nevertheless, genetic variation and molecular epidemiology of *A. fumigatus* from different sources in Iran are underestimated. Hence, the main object of this study was to investigate the molecular epidemiology of clinical and environmental *A. fumigatus* isolates from Iran using microsatellite typing.

## Materials and Methods

### Fungal strains

Sixty-six clinical (n=43) and environmental (n= 23) isolates were obtained from the culture collection of invasive fungi research centre (IFRC), Mazandaran University of Medical Sciences, Sari, Iran. This study was approved by the ethics committee of Mazandaran University of Medical Sciences under the ethics committee code 92-181(2013.4.12). Stock cultures were maintained on malt-extract agar (MEA, Difco, U.S.A.) at 24 °C for one week prior to use. All isolates were collected from six cities of Iran comprising of Mashhad (n=15; 22.7%), Tehran (n=19; 28.7%), Sari (n=18; 27.2%), Shiraz (n=9; 13.6%), Hamadan (n=4; 6%), Babol (1; 1.5%). Clinical isolates comprising bronchoalveolar lavage (n = 29; 67.4%), endotracheal (n = 5; 11.6%), sputum (n = 4; 9.3%), sinus discharge (n = 3; 6.9%), lung biopsy (n = 1; 2.3%) and ear swabs (n = 1; 2.3%), but environmental isolates collected from soil samples surrounding from hospital gardens (n = 14; 60.8%), air (n = 9; 39.1%). All isolates were initially screened by macro- and microscopic features, ability to grow at >45°C and were confirmed to the species level by DNA sequencing of the ß-tubulin as previously described [ 11 ]. Briefly, the fungal mycelia were grown on sabouraud dextrose agar plates. Total DNA was extracted according to the manufacturer’s instructions of Ultra Clean Microbial DNA Isolation Kit (Mobio, U.S.A.) and were stored at −20 °C prior to use [ 24 ]. 

### Microsatellite A. fumigatus typing

To perform microsatellite typing, three distinct multiplex PCRs with a panel of nine short tandem repeats (STR) were designed to evaluate the genetic relationship between the isolates as previously described [ 25 ]. To differentiate the three loci within one multiplex PCR the forward primers were labeled with FAM-, JOE- and HEX-fluorphore at the 5´side. Briefly, PCR assays were implemented in a volume of 25 µl, containing 1 µM of all amplification primers, 1 U of FastStart *Taq* DNA polymerase (Roche Diagnostics), 0.2 mM deoxynucleoside triphosphates and 2µl of target DNA in 1× reaction buffer. Thermocycling was done in a thermocycler (Westburg- Biometra USA ) as described before [ 25 ]. PCR products were diluted 100-fold with ddH2O; subsequently 1 µl of this diluted PCR product was added to 8.9 µl ddH2O and 0.1 µl of CC-500-ROX marker (Promega, Leiden, TheNetherlands). The samples were boiled for 1 min at 100 °C and the fragment sizes were defined using an ABI3500xL Genetic Analyzer platform, afterward (Applied Biosystems, Foster City, CA, USA) according to the manufacturer instructions. The genomic link among *A. fumigatus* strains was determined by comparing the profiles with BioNumerics v6.6 software (Applied Maths, Sint-Martens-Latem, Belgium). To generate the dendrogram, or the option to generate a minimum spanning tree directly from the categorical data, un-weighted paired group method was applied using arithmetic average (UPGMA) algorithm was applied. The Simpson index diversity (*D*) was used to measure the genetic distinction or diversity between *A. fumigatus* isolates. A ‘*D*’ value of 1 indicates that all isolates are different whereas a ‘*D*’ value of 0 indicates that all isolates are identical [ 26 , 27 ].

## Results

**Figure 1:**
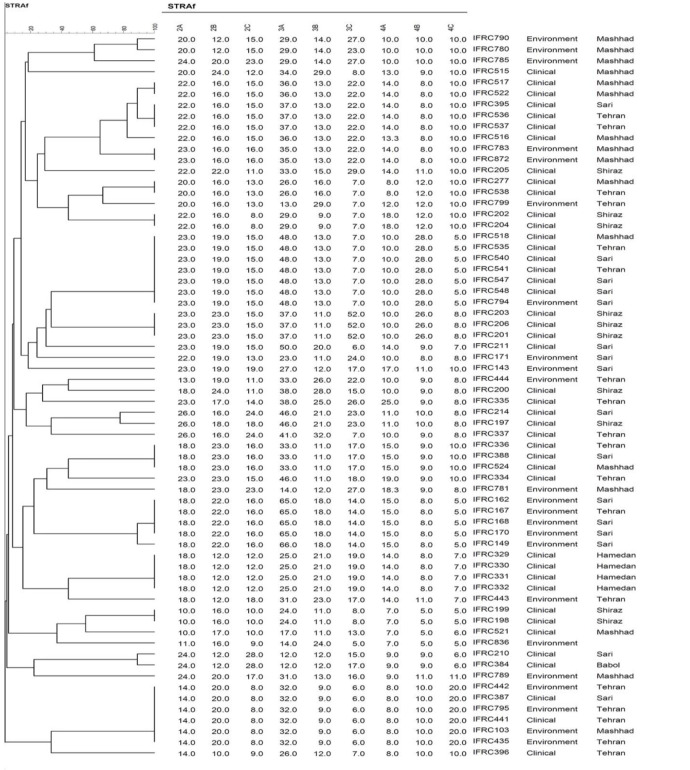
Dendrogram based on profiles of nine STR markers from 66 *A. fumigatus* isolates. The dendrogram is based on a categorical analysis of 9 microsatellite markers in combination with UPGMA clustering. The scale bar above the dendrogram indicates the percentage identity between the genotypes.

**Figure 2:**
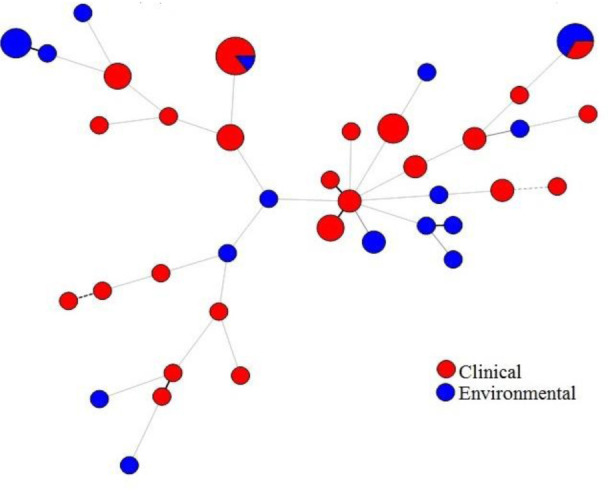
Minimum spanning tree (MST) representing the genotypic diversity of 66 clinical and environmental *A. fumigatus* isolates using microsatellite typing.

**Figure 3:**
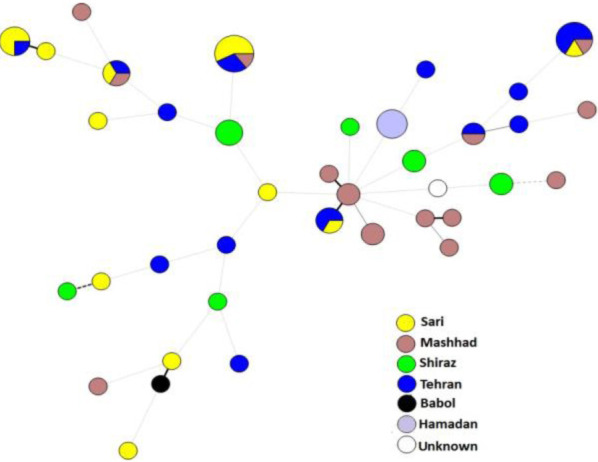
Minimum spanning tree (MST) representing the genotypic diversity of 66 *A. fumigatus* isolates from Iran using microsatellite typing.

**Figure 4:**
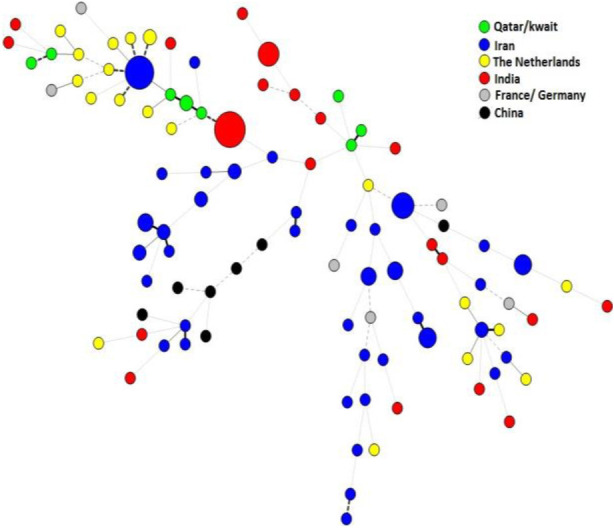
Minimum spanning tree representing the genotypic diversity of *A. fumigatus* isolates from Iran and those outside Iran.

Sixty-six *A. fumigatus* isolates from different sources were genotyped using the full panel of 9 short tandem repeats markers. The Simpson’s index of diversity was less than 0.9 for all nine markers combined. According to the STR typing, 38 distinct genotypes were distributed among environmental and clinical isolates. Remarkably, table 1 summarized the number of clinical strains which was higher than environmental one; more different genotypes was found in the clinical isolates (n=8) rather than in the environmental one (n=2). Among all genotypes, 26 (68.4%) genotypes were only found once, 5 (13.1%) genotypes were observed 2 times, 3 (7.8%) genotypes were observed 3 times, 2 (5.2%) genotypes were observed 4 times, 1 (2.6%) genotypes were observed 6 times, and 1 (2.6%) genotypes were observed 7 times (Figure 1). In this dendrogram, two of related genotypes could be identified differing only at a single locus. Twelve clones comprising 40 different isolates and representing 60% of all isolates were identified. Each clone included 2–7 isolates. According to genotyping results two isolates from soil samples and one clinical isolates from BAL sample of hospitalized patient were identical. We identified a clonal cluster including 6 (4 environmental and 2 clinical isolates), azole resistant isolates harboring cyp51A gene mutations. The three environmental strains of this cluster were concurrently isolated from Tehran's’ hospitals, one isolated from a patient hospitalized in Tehran, one isolated from a patient hospitalized in Sari, and one isolate originated from environment of hospital in Mashhad city (Figure 1). Figure 2 illustrates the genotypic diversity distribution of the environmental and clinical *A. fumigatus* isolates. Eight genotype clusters included only clinical isolates, two cluster only environmental isolates and two clusters contained clinical and environmental isolates. Figure 3 show the geographically diverse *A. fumigatus* isolates. The STR typing depicted no genotypic correlation of Iranian *A. fumigatus* with isolates from other countries. In addition, the high genetic diversity observed of Iranian *A. fumigatus* isolates with those outside Iran and formed a separate cluster (Figure 4).

**Table1:** *A. fumigatus* genotypes found in environmental and clinical samples

Sample source	No. of isolates	Niche and number of isolates	No. of different genotypes	No. of same genotypes
Environmental	23	hospital gardens:14	2	2
		Air: 9		
Clinical	43	BAL: 29	8	
		Endotracheal: 5		
		Sputum: 4		
		sinus discharge:3		
		lung biopsy: 1		
		ear swabs: 1		

The number of allelic mismatches among STR profiles was used as distance. Each circle represents a unique genotype (Gt). The size of the circle is correlated with the number of isolates possessing the corresponding Gt. Dark, dashed and thin connecting bars corresponds to one, 2 or >2 different markers observed between linked Gt. Gts with a shaded background contain a minimum of 2 isolates that differ maximum in 1 microsatellite marker as the possible result of microevolutionary events and are likely to be clonally related. 

The image shows that some *A. fumigatus* isolates from different cities were shared in a cluster. Among different genotypes, 6 clusters comprised isolates of different cities.

The image shows that the high genetic diversity observed of Iranian *A. fumigatus* isolates with those outside Iran and formed a separate cluster.

## Discussion

*A. fumigatus* is globally found in the hospital environments and most frequently isolated from patients with IA or aspergilloma [ 28 - 34 ]. the highest airborne *Aspergillus* conidia concentrations due to the construction inside or surroundings area of the hospitals as well as problems in the ventilation or conditioner systems have proposed the environment to be the potential source of infection [ 35 , 36 ]. Definitely, monitoring the genotype of the isolates is the only way to determine that airborne conidia have caused the infection [ 37 ]. Several molecular typing techniques have been extended in order to understand epidemiological relationships between environmental and clinical isolates obtained from various origins to more specifically demonstrate the source of disorder [ 38 ]. In several studies, various methods have been performed for *Aspergillus* genotyping, but [ 39 ], they lack the essential reproducibility between experiments [ 40 , 41 ]. de Valk *et al*, newly described a novel panel of 9 STRs for genotyping of *A. fumigatus* with highly discriminatory power, clear assignment, inter-laboratory exchangeability of the results [ 25 ]. However, it has rarely been used for epidemiological study of IA outbreaks. Kidd *et al*. [ 42 ] and Balajee *et al*. [ 43 ] designed an epidemiological study of invasive aspergillosis outbreaks in hospitals wards and validated STR*Af* as a main genotyping tool. Unlike other *Aspergillus* species, there is little information about the genotypic diversity of *A. fumigatus* from different sources in Iran [ 44 ]. Here, it was revealed the high genotypic variability among Iranian *A. fumigatus* isolates. Hence, 38 distinct genotypes were identified within 66 Iranian *A. fumigatus* isolates using a panel of nine microsatellite markers. The large number of genotypes was in concordances with the results of other studies [ 45 , 46 ]. Unlike Bart-Delabesse *et al*., we were able to two cluster isolates by their clinical or environmental origin [ 46 ]. Our study confirmed results obtained by de Valk *et al*. reported that majority of the patients were affected with only one genotype [ 23 ]. This observation suggesting a common environmental source in these patients and it can lead to new approaches to infection control to prevent aspergillosis in immunocom-promised patient. In our pervious study, genotyping analysis identified that 41 out of 44 *A. fumigatus* strains with the TR34/L98H mutation, isolated from compost in 13 different Iranian cities, shared the same allele across all nine examined microsatellite loci [ 22 ]. Like our finding, Chowdhary et al. illustrated a clonal spread and emergence of environmental azole resistant *A. fumigatus* isolates from different parts of India. This strains shared the same genotype not found in any other evaluated samples within or outside of India [ 47 ].

## Conclusion

The STR typing is considered as a valuable tool with excellent discriminatory power to study the molecular epidemiology and genotypic diversity of *A. fumigatus* isolates. Generally, these findings show that the high genetic diversity observed of Iranian *A. fumigatus* isolates with those outside Iran and formed a separate cluster.

## Authors’ contribution

H. B., T. SH and M. N., conceived the study. M. N., M. M, and M. F prepared sampling and performed molecular identification and analyzed the data. S. Kh performed Microsatellite assay and H.B, M.N interpreted the data. H. B., T. SH, S. Kh and M. N prepared the manuscript. All authors approved the final version of the manuscript.

## Financial disclosure

No financial interests related to the material of this manuscript have been declared.
